# The Need of an Intermediate Single-Room Service in Hospitals
*Paper read at the twelfth annual conference of the American Hospital Association, held at St. Louis, Mo., September 1910.


**Published:** 1910-12-03

**Authors:** C. Irving Fisher

**Affiliations:** Superintendent, the Presbyterian Hospital in the City of New York.


### SPECIAL INSTITUTIONAL ARTICLES.

### THE NEED OF AN INTERMEDIATE SINGLE-ROOM SERVICE IN HOSPITALS.*

By C. IRVING FISHER, M.D., Superintendent, the Presbyterian Hospital in the City of New York.

Our great institutions are complex organisms which have grown up from small beginnings, enlarging step by step along the lines of human needs. These needs are ever growing and modifying. The test of the real live worth of any institution is its adaptability to the present, its ready modification to changing states of society, its power to grow.

The tracing of the past development of any institution is an interesting study for the philosophic mind. The looking forward into the probable and possible future development appeals to the imaginative and prophetic. But the practical worker at every step must ask the practical question, "What is the unfilled need to-day? What shall be the shaping of the next advance step that this need also may be served?"

### The Beginning of Hospitals.

In its inception the hospital was a charity. It was established to take pity on the sick and homeless poor, and was simply a poor makeshift as a last resort for the helpless and forlorn. As such, we read of houses of detention in connection with the temples of many religions long before the Christian era. As such it continued its career through the centuries of the conflict between Christianity and Mohammedanism and the Middle-Age struggle between Protestantism and Catholicism. This initial idea still persists, and must persist, as a prominent feature of our now many-sided and ever-broadening work.

Within fifty years hospitals have multiplied in the land, but there are now few homeless wanderers, and for these there are provided institutions at public charge. They are practically eliminated from the problem of our hospitals supported by private funds.

The many hospitals either endowed or supported by voluntary contributions, while they refuse aid to none, have now their real reason for being in the needs of the independent, self-respecting, self-supporting community with their families, which is the real citizenship. These naturally divide themselves into three groups.

### Hospitals for the Indigent Poor.

First, the really poor—those who, struggling for self-support against adverse conditions, either in their own make-up or from external circumstances, are barely able to make the ends meet, who are at best but little above actual poverty, and who when sickness comes are without resources. These include most of the great army of unskilled workers, whose wages are too small for a really comfortable living, even when health and the times are good. The hospital as a charity is for these, and it is primarily with the care of these that our hospitals have in the more recent past been occupied. It is with these that our wards are for the most part filled. Among these are grades of thrift and ability to pay, and so we establish nominal ward rates, while all, whether able to pay or not, receive the same care, conditions, and service. In its provision for these the hospital of to-day seems to be so perfected as to be nearly beyond criticism. The only hardships which the ward imposes are lack of privacy, the chance of agreeable or disagreeable neighbours, in convalescence a diet arranged for health-building rather than catering to individual taste, and the inevitable movements in the open ward at nights. To such things as these, however, the average patient in the ward is already well accustomed. There is also in the ward a necessary limit set upon the visits of friends, due to consideration of the best good of all.

This service of the hospital for the very poor, with which the hospital started, must continue as one of its features to the end. "The poor always ye have with you." Already, however, new elements of immense value to the community have come in, and an ever-widening vista of possibilities is opening to the hospital worker who is also a student of social need.

### The Influence of Schools.

The first step came in the direction of research and education. Medical and surgical science discovered within the hospitals the best opportunity for broad and comparative observation and experiment, and medical students, both undergraduate and graduate, flocked to them for original study and investigation. They became the centres for the marvellous discoveries which have within less than half a century completely revolutionised diagnosis and treatment. Schools for the training of nurses were added, and nursing became an art and a profession. At length the public began to discover that in these institutions were collected the best knowledge and skill of the age, with all needed supplies for all manner of treatment, such as no private physician's office could afford, no private home, however wealthy, could supply.

Then those of greatest wealth in the community began to knock at the doors for admission, saying, "We want the best, and we will pay well for it." It dawned upon the minds of the hospital managers that by establishing private rooms and imposing a suitable price for their use an income might be derived towards the operating expenses. To these private rooms the skilled physicians and surgeons, who had already connected themselves with the hospital for purposes of investigation, now began to bring their wealthy patients, still collecting from them their full private fee, while the hospital also received the price it had set upon the room, with supplies, service of nurses, and all necessary accessories.

* Paper read at the twelfth annual conference of the American Hospital Association, held at St. Louis, Mo., September 1910.

### Ward and Private Room Service.

These two classes of service—the ward service and the private room service—have become well established and proved their value. But the beneficiaries of the one and the patrons of the other do not constitute the great bulk nor the most important part of the community. There remains the so-called " middle class " as gauged by money standards, but by the standards of usefulness the most necessary and normal part of the body politic, independent-spirited, willing to suffer, but not willing to compromise with self-respect. This group includes most of the brain and brawn on which the very life of the State depends: the skilled artisans and mechanics, small merchants, literary people, artists, teachers, ministers, and the great army of salaried people, with the families to whom these belong. They do not ask for charity and do not wish to receive it. They lay by for the rainy day, and when the rainy day comes they want to meet it with independence and self-respect. They do not live when well on the same scale with wealth, and they cannot afford to be sick on that scale either. Their tastes and desires are moderate; they do not demand luxurious surroundings nor unseasonable dainties. But they want in sickness that to which they have been accustomed in health, a degree of privacy and personal choice of companionship which the open ward does not admit, and the satisfaction which the genuine soul finds in paying its own way with the product of its own industry and thrift. For these, up to date, there has been no definitely planned or adequate provision. Some of the hospitals in the smaller towns have arrangements which more nearly meet their need, but in our city hospitals the private room is beyond their limit; to enter the ward would, in their own eyes and in the eyes of their neighbours, put them at once into a strata of society to which they do not belong and impose conditions they cannot face. So they and their families shoulder the burdens of sickness in their own homes under conditions far more limited than those of more primitive days, when people were not so crowded together in small apartments, and when home treatment was universal and so better understood.

### Hospitals as Factors in Preventing Waste.

To provide an intermediate service for these, the great bulk and bulwark of the community, would seem to be both good philanthropy and good economics. From the standpoint of philanthropy, why should they be left to carry burdens and suffering from which both the very poor and the very rich have been relieved? From the standpoint of social economics, if our aim is singly to serve the best interest of the public, how can we afford to withhold from these the best chances for quick and complete recovery—to allow such loss of human energy and productive power by the prolonged periods of sickness and convalescence, the often untimely end, and the always enormous waste which results when bread-winners and economic producers must drop out to care for sick friends, and the whole family strength is turned from its normal channel to the heavy and often hopeless task of nursing without adequate supplies, knowledge, or the skill which comes from experience? Moreover, the loss of efficiency and productive power involved is not confined to the immediate family of the patient, but sends out waves of disturbance far into the surrounding community, as will be readily seen by tracing one specific case out into all its secondary effects. The hospital is *the* agent best fitted to check this immense and most lamentable waste.

For ages men have recognised the philanthropic side of hospital service; for many decades they have recognised the educational side; but they are only discovering the economic side and what it might be made to accomplish.

### The Private Room System.

Having recognised the need, how shall it be met? Plain single rooms or very small wards, with a service a little better or even identical with the ward service, would give privacy and make possible larger privileges in visits from friends, and a fee could be charged in proper proportion. This fee, of course, would have to be determined by each hospital independently, according to the scale of prices which prevail in the outside community which this particular hospital serves. There might be very properly added a moderate fixed fee to physician or surgeon for his attendance, corresponding with the regular charge of the moderate-priced doctors whom this order of patients are able to employ outside. These patients might also be allowed some choice or preference as to which doctor or surgeon already on the hospital visiting board should attend them.

### Visiting Nurses.

A system of visiting nurses with hospital backing, and for whose services a moderate fee should be charged, payable to the hospital, would serve this class of people much more satisfactorily than any system now generally in use; but, on the other hand, most city dwellers live in apartments too small to make a quiet sick-room possible; the amount of strength drawn from the other members of the family by the mere presence of severe sickness in the home can ill be spared by these busy people, who are already shouldering the bulk of the world's work; while the work of the nurses is made more difficult, the doctor's treatment often neutralised, and the chances of quick recovery often lessened by the effect on the patient of the mutual sympathies of the patient and the friends in the home.

It should not be overlooked also that out of such kindly and just consideration there will come, in the long run, a great economic advantage to the hospital itself. Many of these will afterwards gratefully contribute to the hospital the " many a little " which " makes the mickle." Moreover, out of this thrifty and substantial portion of the community will come the prosperous and wealthy who will be the future friends and supporters of the hospital, the more surely and firmly because they have experienced its benefits and know of themselves its value. The patient who accepts the charitable service of the ward seldom becomes a contributor.

Wanted—Education.

The physicians, probably more than the managers, need to be educated for this form of service. We have heard much of dispensary and hospital abuses, but it is the frequent attitude of the doctors toward these people of limited means that, more than anything else, has been driving them to accept the free help of dispensaries. Not long ago I called the attention of one of the leading specialists connected with our dispensary to a young woman, a teacher, who needed care and who could pay $2 or $3, but could not afford to pay his regular office fee of $5, and I asked if he would see her at his office. His reply was, "No; if she cannot pay my fee, I would rather treat her here." It ought not to have taken that patient long to see that for her it was much simpler to get skilled advice and treatment *without* pay than to obtain it for the fee which came within her means. There are plenty of bright, capable younger men in the profession who need practice and would gladly have treated such a patient at her own price, but of these the great man said nothing. The doctor who has worked his way up to the point in his profession where he can command the large fees of the wealthy is quite justified in reserving his time and energies for those who can pay him lavishly, and justified also in maintaining in his office as exclusive a tone as his wealthy patrons desire. But no man should be retained on the board for any part of the hospital service who has not some insight into the true hospital spirit. There should be no room on hospital boards for those eminent physicians and surgeons who accept the position chiefly because the private room is a convenient place where they may send their wealthy patients, with house staff and nurses to relieve them of worry and responsibility; nor for those who enter the hospital service solely because of the chance it gives them to see and study a great variety of interesting cases; nor for those who have so little regard for the true interests at stake that they will, either willingly or through indifference, pauperise a self-respecting person of small means by turning him over to the free list instead of taking pains to direct him to good moderate-priced service. The patient desiring treatment has not the data necessary for intelligent selection of such service. He must turn for direction either to the man of large public prominence and well-known repute in the profession or to the hospital, and if these fail unselfishly to advise him in the direction of his best interests he is in bad case indeed. We have much reason to believe, however, that this is too often so.

The Example of the Mayo Brothers.

One of the elements of the fame and success of the well-known Mayo Brothers, of Rochester, Minn., has, I understand, been due to the fact that patients' circumstances have been investigated very strictly by honest business methods, and a scale of fees established which is fair to all concerned. Certainly such methods are possible with all hospitals.

This service could not, of course, be made to cover its full expense to the hospital, neither should it be regarded as in any sense a charity. The added cost should be charged to the account of public benefit, aiming to conserve to the community as much as possible of its valuable producing power in the person of its chief workers.

Some Details.

In instituting this intermediate service it will be necessary to place about it such regulations as will safeguard it from any effort of the parsimonious rich to obtain use of these rooms, or of attending physicians and surgeons to put into them their wealthy patients who should pay the price of the more expensive service. The fees for these rooms should be very moderate and definitely fixed, and it might be desirable to have an enlarged corps of attendings, making it include more of the younger rising men of real worth and true hospital spirit. It might even prove eventually expedient to have distinct though closely co-operative branches of the visiting board for the different branches of the service, while above all should stand a board of consultants only, composed of men who were advanced from the boards of attendings.

All such details would have to be worked out by each hospital for itself with various experiments and frequent modifications, as the expediency or inexpediency of details has been tested out in every other part of the service.

